# Antimicrobial susceptibility test and antimicrobial resistance gene detection of extracellular enzyme bacteria isolated from tilapia (*Oreochromis niloticus*) for probiotic candidates

**DOI:** 10.14202/vetworld.2023.264-271

**Published:** 2023-02-11

**Authors:** Mira Mawardi, Agustin Indrawati, I. Wayan Teguh Wibawan, Angela Mariana Lusiastuti

**Affiliations:** 1 School of Veterinary Medicine and Biomedical Sciences (SVMBS), IPB University, Jl. Agatis Kampus IPB Dramaga Bogor, 16680, Indonesia; 2 Main Center for Freshwater Aquaculture, Ministry of Marine Affairs and Fisheries, Jl. Selabintana No. 37, Selabatu, Kec, Cikole, Kota Sukabumi, Jawa Barat 43114, Indonesia; 3 Research Center for Veterinary Sciences, National Research and Innovation Agency, RE Martadinata 30 Bogor, Jawa Barat, Indonesia

**Keywords:** antibiotic, antimicrobial resistance, enzymatic bacteria, *Oreochromis niloticus*, probiotic

## Abstract

**Background and Aim:**

Antimicrobial resistance (AMR) is a global problem that can increase mortality and morbidity rates and adversely affect health. Therefore, AMR control must be carried out in various sectors, including the fisheries sector, using probiotics. Bacteria can become resistant to antibiotics, including bacteria used for probiotics. This study aimed to isolate bacteria as potential producers of extracellular enzymes, phenotypic characterization, and antibiotic-resistant gene patterns.

**Materials and Methods:**

In this study, 459 bacterial isolates were isolated from the stomach of tilapia in Indonesia. Tilapia was obtained from Sukabumi, Ciamis, Serang, Banjarnegara, Jayapura, Sorong, Manokwari Selatan, Takalar, Lampung, Batam, and Mandiangin. Enzymatic bacteria were identified. An antimicrobial susceptibility test was conducted by agar disk diffusion, and genotypic detection of encoding genes was performed using a molecular method.

**Results:**

This study obtained 137 isolates (29.84%) that can produce extracellular enzymes. The highest number of E-sensitive isolates was found, including 130 isolates (94.89%). Six isolates (6/137) can produce four enzymes (amylase, protease, cellulose, and lipase), and they were sensitive to antibiotics. A total of 99 isolates can produce extracellular enzymes, and they were sensitive to antibiotics. Such isolates serve as a consortium of probiotic candidates. The isolates that are resistant to oxytetracycline (OT), erythromycin (E), tetracycline (TE), and enrofloxacin (ENR) included 15 isolates (10.95%), seven isolates (5.11%), three isolates (2.19%), and one isolate (0.73%), respectively. In addition, four isolates (2.92%) were detected as multidrug-resistant. The *tet*(A) gene obtained the highest result of detection of resistance genes in isolates that were intermediate and resistant to TE and OT. Isolates that serve as ENR intermediates have a high *qnr*(S) resistance gene.

**Conclusion:**

The data in this study provide the latest update that bacteria can serve as a consortium of potential probiotics with antibiotic-resistant genes for the treatment of fish. Bacteria that are intermediate to antibiotics may contain resistance genes. The results of this study will improve the policy of probiotic standards in Indonesia.

## Introduction

Aquaculture products are a source of food for human needs. The increase in fish production continues to meet food needs, one of which is intensive fish cultivation with the application of technology to increase and maintain fish production. Freshwater fish such as tilapia are in great demand for local and international consumption; thus, it is suitable for sustainable cultivation in the future [1]. Indonesia is the second major producer of tilapia worldwide after China, accounting for above 50,000 tons in 2018. Indonesia’s production value reaches 1,222,700 tons or 20.27% of global production. Other major producing countries include Egypt, Bangladesh, Brazil, Philippines, Viet Nam, Thailand, Colombia, Ghana, Uganda, Taiwan POC, and Mexico [2]. Indonesia continues to strive to increase the production of tilapia aquaculture because it has a high consumption value. At present, the use of probiotics is considered as a method of fish farming. Probiotics are used for disease prevention and environmental quality control. In addition, probiotics are an expected effector to improve fish health [3]. The use of probiotics in fish farming can increase growth [4–10]. Probiotics can be used for disease prevention, and they can act like antibiotics. Probiotics contain microorganisms that have an impact on fish and the environment [11]. Several types of probiotics can be used from the group of Gram-negative bacteria, Gram-positive bacteria, yeast, bacteriophages, and microalgae. Bacteria that have quorum quenching ability can be potential probiotics, such as those belonging to the family Flavobacteriaceae [12–14].

Antimicrobial resistance (AMR) is considered as a global threat, resulting from the use of drugs and microbial resistance in the environment, which affects human, animal, and environmental health. Antimicrobials play an important role in health, and they are associated with infectious diseases in animals and humans. They can also control the emergence and spread of resistance among pathogenic or commensal bacteria in the environment [15]. Antibiotics greatly affect the aquatic environment, which can increase the sensitivity of antibiotics to microbiota [16]. The presence of microbiota resistance in the environment is also strongly influenced by human activities [17]. Currently, AMR in aquaculture is being developed, and it remains a concern because it is a global animal food source sector experiencing an increase [18].

In this study, the importance of selecting bacteria as probiotic candidates was revealed by conducting antibiotic sensitivity tests because bacteria have the potential for antibiotic resistance, and they contain resistance genes that can be transmitted to other microorganisms in the environment. In addition, the importance of antimicrobial susceptibility tests, particularly on the use of antibiotics in fish, was highlighted. For example, bacteria used as probiotics can potentially become resistant to antibiotics. This research can isolate bacteria as a consortium of probiotic candidates. Probiotic candidates are selected as producers of extracellular enzymes, which are sensitive to antibiotics used for fish in Indonesia. This study aims to isolate and characterize bacteria that can be used as probiotics, to perform phenotypic testing to observe resistance patterns, and to genotype characterization of their encoding genes.

## Materials and Methods

### Ethical approval

This research has been approved by the Committee on Animal Code of Ethics, Faculty of Veterinary Medicine, Bogor Agricultural University, IPB University with the number: 023/KEH/SKE/VIII/2022.

### Study period and location

**Figure-1:**
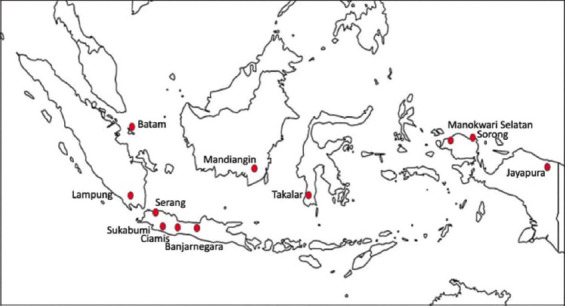
Location sampling of bacteria isolated from tilapia. [Source: https://peta-hd.com/peta-indonesia-hitam-putih/].

The study was conducted from June to September 2022. Tilapia samples were obtained from several fish farming areas in Indonesia (Figure-1), such as Sukabumi, Ciamis, Serang, Banjarnegara, Lampung, Batam, Mandiangin, Takalar, Jayapura, Manokwari Selatan, and Sorong. The samples were processed at Main Center for Freshwater Aquaculture Laboratory, Sukabumi.

### Bacterial inoculation from fish

Fish were collected from cultured fish ponds in accordance with the following criteria: Healthy fish and no ulcer on the body of the fish. The fish were necropsied, and the stomach was removed. The stomach of the fish was split open, and the stomach contents were removed using a sterile scalpel. Then the inside of the stomach wall was rubbed on the surface of the Nutrient Agar (Oxoid, France) media. It was incubated at room temperature (28°C) for 24 h and stored in a refrigerator at 4°C until further tests were performed.

### Isolation and identification

Each colony that grew on nutrient agar media was collected and isolated. The isolates were streaked in four quadrants on nutrient agar media (Oxoid) to obtain pure cultures. The isolates were incubated at 28°C for 24 h. Bacterial identification was conducted using the biochemical analytic profile index (API) 50 CH, API 50 CHB, and API 20E kit (BioMérieux, France). Tests were conducted in accordance with the manufacturer’s instructions. Analysis of the test results was carried out on the software https://apiweb.biomerieux.com/login.

### Characterization of bacteria through extracellular enzyme production

#### Proteolytic activity

Proteolytic bacteria were selected using nutrient agar media (Oxoid) plus skim milk as a protein source. Nutrient agar media (Oxoid) were added with 1% skim milk (HiMedia, India), heated until homogeneous, and boiled. The media were sterilized at 121°C for 15 min. The media were stored at room temperature 28°C for 24 h and then at 4°C until used.

#### Amylolytic activity

Amylolytic bacteria were selected by adding media with carbohydrate sources. The composition of the media per liter consisted of 10 g of corn starch (EgaFood, Indonesia), 4 g of KH_2_PO_4_ (Merck, USA), 2 g of Tryptone (HiMedia), 0.2 g of MgSO_4_.7H_2_O (Merck), 0.001 g of CaCl_2_ (Merck), 0.004 g of FeSO_4_.7H_2_O (Merck), and 15 g of Bacto Agar (HiMedia). The medium was added with water and then heated until it boiled. The media were sterilized at 121°C for 15 min and poured into Petri dishes. The media were placed at room temperature 28°C for 24 h and then stored at 4°C until used.

#### Cellulolytic activities

Cellulolytic bacteria were selected using media with the addition of cellulose, namely, carboxyl methyl cellulose (CMC). The composition of the medium per liter was 10 g of CMC (HiMedia), 4 g of KH_2_PO_4_ (Merck), 4 g of Na_2_HPO_4_ (Merck), 2 g of Tryptone (HiMedia), 0.2 g of MgSO_4_.7H_2_O (Merck), 0.001 g of CaCl_2_ (Merck), 0.004 g of FeSO_4_.7H_2_O (Merck), and 15 g of Bacto Agar (HiMedia) [19]. The media were added with water, stirred with a magnetic stirrer until homogeneous, and then heated until it boiled. The media were sterilized at 121°C for 15 min and poured into a Petri dish. The media were placed at room temperature 28°C for 24 h and then stored at 4°C until used.

Staining to see the hydrolytic zone: 5 mL of Congo red (Central Drug House, India) 0.5% was poured on the surface of the media and incubated at room temperature 28°C for 15 min. Congo red solution was removed from the media, added with 5 mL of 2M NaCl (Merck) solution, and then incubated for 15 min at room temperature 28°C. Next, 2M NaCl solution was removed from the media, and the media were dried. The zone of inhibition formed around the disk was observed.

#### Lipolytic activity

Lipolytic bacteria were selected using tween agar. The composition per liter consisted of 10 g of peptone (HiMedia), 5 g of NaCl (Merck), 0.1 g of CaCl_2_.2H_2_O (Merck), 20 g of Bacto Agar (HiMedia), and 10 g of Tween 80 (Merck) [20]. The medium was added with water and then heated until it boiled. The media were sterilized at 121°C for 15 min and then poured into a Petri dish. Afterward, the media were placed at room temperature 28°C for 24 h and then stored at 4°C until used.

#### Preparation of bacteria

Bacteria were cultured on nutrient broth media at 28°C for 24 h. Then, 10 μL of the bacterial solution was dropped on a blank disk (Oxoid) that had been placed on agar media. Samples were incubated at 28°C for 24–72 h. The inhibition zone formed around the disk was measured using an electronic digital caliper (mm).

#### Antimicrobial activities

The antibiotic disks (Oxoid, USA) used in this study included 30 μg of tetracycline (TE), 30 μg of oxytetracycline (OT) (or intermediate to TE [OT], 5 μg of enrofloxacin [ENR], and 15 μg of erythromycin [E]). Bacterial isolates with a concentration equivalent to a turbidity of 0.5 McFarland were inoculated on Mueller-Hinton agar (Sigma, USA) media using a sterile collection swab (Citoswab, France). Samples were incubated at 35°C for 24 h. The isolates were phenotypically characterized by measuring the inhibitory zone formed around the disk, and the results were interpreted in accordance with the Clinical and Laboratory Standard Institute for Enterobacter [21].

#### Antimicrobial-resistant gene detection

Samples of one bacterial colony were added with 1000 μL of sterile ddH_2_0 and heated at 95°C for 5 min. The sample was centrifuged at 14,534× g and 4°C for 15 min. In addition, 500 μL of supernatant was transferred to a sterile tube and stored at −20°C as a DNA sample for amplification. Amplification was performed using a final reaction volume of 25 μL, consisting of 12.5 μL of GoTaq^®^Green (Promega, USA), each 0.5 μL of forward primer and reverse primer, 9.5 μL of sterile distilled water, and 2 μL of DNA samples. The thermal cycle used a denaturation temperature of 95°C for 2 min; 40 temperature cycles of 95°C for 30 s, annealing (Table-1) [22–25] for 30 s, and 72°C for 1 minute; and final extension at 72°C for 5 min. The primer pairs (Primer Integrated DNA Technologies, Singapura) used in this study included *tet*(A), *tet*(B), and *tet*(E) for TE; *erm*(B) for macrolide; and *qnr*(S) for fluoroquinolone. The amplification results were electrophoresed in 1.5% agarose (Vivantis, Malaysia) with TAE (Vivantis) at 100 voltage for 10 min. The electrophoresis results were observed on UV electrophoresis gel.

**Table-1:** Primer pairs used for detection-resistant genes.

Genes target	Sequences (5′–3′) class (Forward-reverse)	Annealing temperature (°C)	Amplicon size (bp)	References
*tetA*	GGTTCACTCGAACGACGTCA	60	577	[22]
	CTGTCCGACAAGTTGCATGA			
*tetB*	TACGTGAATTTATTGCTTCGG	60	206	[23]
	ATACAGCATCCAAAGCGCAC			
*tetE*	GGTATTACGGGAGTTTGTTGG	60	199	[23]
	AATACAACACCCACACTACGC			
*ermB*	GAAAAGGTACTCAACCAAATA	55	639	[24]
	AGTAACGGTACTTAAATTGTTTAC			
*qnrS*	ACGACATTCGTCAACTGCAA	55	417	[25]
	TAAATTGGCACCCTGTAGGC			

## Results

**Figure-2:**
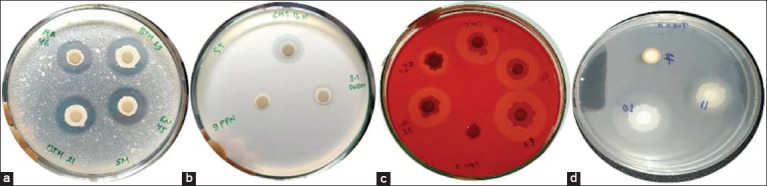
Zone diameter of isolated bacteria (mm) (a) proteolytic, (b) amylolytic, and (c) cellulolytic is the diameter zone seen after incubation and then stained with Congo red (d) lipolytic activities.

The bacteria were isolated from the stomach of healthy tilapia, and no wounds were observed on the body of the fish. These criteria were selected to obtain non-pathogenic bacteria. The results of this study obtained as many as 459 isolates from various locations (Table-2). Then, each isolate was characterized phenotypically as a producer of extracellular enzymes, namely, protease, amylase, cellulose, or lipase (Figure-2). The characterization results have obtained 137 isolates, and they can produce extracellular enzymes. The highest number of bacteria as producers of extracellular enzymes obtained from the Banjarnegara location was 33 isolates from 137 isolates.

**Table-2:** Number of bacterial isolates and extracellular enzyme-producing isolates from each location.

Location	Total isolate	Extracellular enzymes (isolate)	Extracellular enzymes (%)
Ciamis	42	13	31.0
Sukabumi	40	16	40.0
Sorong	11	9	81.8
Manokwari Selatan	15	4	26.7
Jayapura	14	3	21.4
Serang	48	4	8.3
Takalar	55	9	16.4
Lampung	65	6	9.2
Batam	52	10	19.2
Mandiangin	53	30	56.6
Banjaranegara	64	33	51.6
Total	459	137	

**Figure-3:**
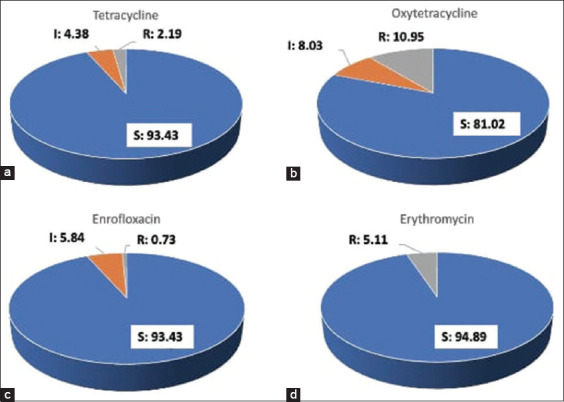
Percentage of antimicrobial susceptibility test on isolates producing extracellular enzymes (137 isolates). (a) Tetracycline, (b) Oxytetracycline, (c) Enrofloxacin, and (d) Erythromycin. S = sensitive, I = intermediate, and R = resistance.

Bacterial isolates as producers of extracellular enzymes were characterized by producing as many as 137 isolates and then tested for antimicrobial activity against antibiotics TE, OT, ENR, and E (Figure-3). A total of 130, 128, 128, and 111 isolates are sensitive to E, ENR, TE, and OT, respectively. In addition, 11, 6, 8, and 0 isolates are intermediate to OT, TE, ENR, and E, respectively. The results of the antibiotic intermediates were categorized as isolates that could not be used as candidates for the probiotic consortium. A number of resistant isolates (15, 10.95%) were obtained against OT. Seven isolates (5.11%), three isolates (2.19%), and one isolate (0.73%) were resistant to antibiotics E, TE, and ENR. In addition, several isolates were resistant to several antibiotics, namely, isolates from Takalar (T1.4) resistant to OT and E, and TE intermediates have been identified as *Aeromonas* spp. The isolate from Mandiangin (MA12) was resistant to TE and OT. The PPN22 isolate was resistant to TE and OT antibiotics and ENR intermediates. The PPN28 isolate that is resistant to three antibiotics, namely, TE, OT, and ENR, was identified as *Aeromonas* spp. Isolates resistant to OT and intermediate to TE were found in isolates SKM8 and SKM13, which were identified as *Aeromonas hydrophila*. Isolates T1-18 and T1-19 were resistant to E and intermediate to TE, and they were identified as *Pseudomonas aeruginosa*.

Based on the results of the characterization of bacteria that produce extracellular enzymes and are also sensitive to antibiotics, bacteria are considered as probiotic candidates. The number of isolates of bacteria that produce protease, amylase, lipase, and cellulose from the total number of isolates has been obtained: 90/137 isolates (65.69%), 46/137 isolates (33.58%), 22/137 isolates (16.06%), and 21/137 isolates (15.33%). Proteolytic bacteria were the most common bacteria, and lipolytic bacteria were the least common. Bacteria that can produce more than one extracellular enzyme (multiple enzymatic) have obtained as many as 50 isolates. Six bacterial isolates can produce amylase, protease, cellulose, and lipase enzymes, including SRG32 for *Bacillus subtilis/amyloliquefaciens*; BTM29 for *Bacillus cereus* 2; and BTM21, BN45, and PPN10 for *Bacillus mycoides*. MA46 refers to *Bacillus firmus*.

**Figure-4:**
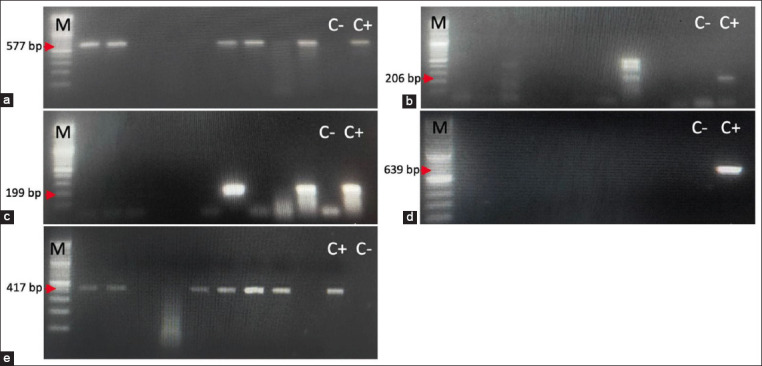
Agarose profile for the detection of resistance gene (a) *tet*A is shown at 577 bp; (b) *tet*B is shown at 206 bp; (c) *tet*E is shown at 199 bp; (d) *erm*B is shown at 639 bp, and (e) *qnr*S is shown at 417 bp. DNA ladder marker 100 bp (Vivantis), C− negative control, C+ positive control.

Then, the results of intermediate and antibiotic-resistant isolates were continuously investigated through genotyping test for resistance genes. The positive results of the detection of resistance genes (Figure-4) using *tet*(A), *tet*(B), and *tet*(E) primers on TE intermediate isolates accounted for (4/6) 66.67%, (2/6) 33.33%, and (2/6) 33.33%. TE-resistant isolates accounted for (1/3) 33.33%, containing the *tet*(A) gene and negative for the *tet*(B) and *tet*(E) genes. The positive results of the detection of resistance genes using *tet*(A), *tet*(B), and *tet*(E) primers on OT intermediate isolates accounted for (7/11) 63.64%, (3/11) 27.27%, and (1/11) 9.09%. OT-resistant isolates were identified, 46.67% (7/15) of which contained the *tet*(A) gene, 13.33% (2/15) contained the *tet*(E) gene, and negative contained the *tet*(B) gene. The positive result of detection of resistance genes using *qnr*(S) primer on ENR intermediate isolate was (6/8) 75.00%. The results of the detection of resistance genes using *erm*(B) primers on seven isolates of resistance E were negative. Several isolates have been detected to have two resistance genes, *tet*(A) and *tet*(B), including isolates MA43, MA44, MA48, T1-18, T1-19, and SKM10.2. The *tet*(A) and *tet*(E) genes were detected in SKM13 and SKM8 isolates. The BN43 isolate was detected by the *tet*(A) and *qnr*(S) genes, which resulted in the antibiotic phenotype being intermediate to OT and ENR. Based on the results of genotypic identification of resistance genes, isolates of antibiotic intermediates have a high chance of detecting resistance genes.

## Discussion

Sampling was conducted from several locations in Indonesia to obtain the best bacterial candidate consortium for probiotics. Microbes in the digestive tract can be influenced by environmental and physiological factors as well as food [26, 27]. Microbes in the digestive tract of tilapia have shown similarities to sediment and water [28]. The abundance of pathogenic bacteria in the digestive tract of sick fish is higher than that of healthy fish, and the difference in bacteria in the digestive tract of sick fish is lower than that of healthy fish [29]. The composition of microbes in the digestion of fish is also influenced by the environment, food, and fish age [30].

The isolation and selection ability of bacteria to produce extracellular enzymes to degrade protein, polysaccharides, and fats have been investigated in tilapia obtained from several regions in Indonesia. Microbial enzymes have catalytic activity and are more stable than enzymes derived from plants and animals [31]. Extracellular enzymes can be produced from microorganisms, including bacteria such as *Bacillus smithii* [32], *Bacillus firmus*, *Micrococcus luteus*, *Bacillus* spp., *Pantoea* spp., *Bacillus ginsengihumi*, *Cellulosimicrobium cellulans*, *Pantoea vagans*, and *Bacillus thuringiensis* [33]. Proteolytic and lipolytic bacteria are widely used and isolated from bacteria because of their broad substrate specificity and higher stability. These bacteria can serve as a potential industry and solve environmental problems [34]. The use of bacteria as an extracellular enzyme producer has been widely used in various industries. Cellulolytic bacteria can be used for cellulose degradation, predominantly found in the *Pseudomonas* group [35]. Thus, such bacteria can be used for waste management through vermicomposting techniques, such as *Acinetobacter baumannii* [36], which can be used in the bioconversion of plants into compost [37]. Cellulolytic bacteria under aerobic and anaerobic conditions were identified from the gastrointestinal tract of *Psammotermes hypostoma* using the CMC agar plate method and Congo red staining [38]. Based on the extracellular ability of enzymes from bacteria, they can be utilized to degrade polysaccharides, proteins, and fats in aquaculture environments. *Bacillus tequilensis*, *Bacillus infantis*, *Bacillus flexus*, *Bacillus paramycoides*, *Bacillus* spp., and *Staphylococcus cohnii* subsp. *urealyticus* can produce the best lipase enzymes [39].

The use of bacteria as a producer of extracellular enzymes is related to the presence of antibiotic resistance and resistance genes. The utilization of bacteria derived from antibiotic-resistant bacteria poses a risk to fish, environment, and human health. Therefore, bacteria as probiotic candidates that are sensitive to antibiotics must be selected as a probiotic consortium of fish, particularly for antibiotic use in aquaculture. Bacterial resistance to antibiotics in aquaculture can be detected in fish and the environment, including water and sediment [40, 41]. Mobile genetic elements, including AMR, play a role in the adaptation and evolution of bacteria [42]. Therefore, bacterial resistance to antibiotics is possible in the cultivation environment. The characterization of bacteria that can produce extracellular enzymes that will be used as probiotic candidates was collected from bacterial isolates sensitive to TE, OT, ENR, and E antibiotics.

Antibiotics used are in accordance with those used for the treatment of fish in Indonesia, including TE (chlortetracycline, OT, and TE), macrolides (E), fluoroquinolones (ENR), and sulfonamides (sulfadiazine) [43]. However, the currently available antibiotics for fish in Indonesia include TE, OT, ENR, and E, which are used for antimicrobial susceptibility tests to obtain candidate isolates of probiotics that are sensitive to these antibiotics. The results of the antimicrobial susceptibility test could obtain probiotic bacteria candidates sensitive to TE, OT, ENR, and E; thus, they are safe to use for fish. Bacteria are sensitive to certain antibiotics, indicating their resistance to these antibiotics. Therefore, the use of antibiotics for treatment is effective. The transmission of resistance genes that can occur horizontally and vertically between bacteria through phages, namely, transduction, conjugation, and transformation [44], will be avoided. Bacteria that are resistant to antibiotics, particularly multi-resistant Gram-positive or negative bacteria, are likely observed because of horizontal gene transfer of resistance genes [45]. Resistant bacteria in aquatic animals can be transmitted to humans through direct contact or food [46, 47]. Probiotic bacteria are resistant to antibiotics used for fish treatment, which will be unsafe. The potential for treatment failure will occur in fish because the infecting bacteria can become resistant, where the resistance gene comes from the probiotic bacteria used. In addition, the potential for contamination of resistance genes in the environment will become uncontrollable, originating from resistant probiotic bacteria capable of breeding in the environment and transferring resistance genes to other bacteria in the environment. Therefore, the selection of a consortium of probiotic candidates must be performed based on antimicrobial activity, particularly for antibiotics used for treating fish.

Based on the results of this study, several potential bacteria were found as candidates for the best probiotic consortium of fish as producers of extracellular enzymes and were sensitive to antibiotics used for fish treatment. A total of 99 isolates out of 137 produced extracellular enzymes, and they were sensitive to TE, OT, ENR, and E. A total of 38 isolates out of 137 isolates were resistant and/or intermediate to TE, OT, ENR, and E or multidrug-resistant. Thus, they could not be used as a consortium of candidate probiotics.

## Conclusion

This study indicated that the potential bacteria as producers of extracellular enzymes that could be used as candidates for the probiotic consortium were detected to be antibiotic-resistant; TE, OT, ENR, E, and multidrug resistance were found. Bacterial isolates can be used as extracellular enzymes and candidates for probiotic consortium, and they are sensitive to antibiotics. Probiotics that contain bacteria that are not resistant or intermediate to antibiotics can be applied for the future safety of aquaculture and the environment. Future studies must include pathogenesis testing, acid resistance, bile testing, and the application of a consortium of probiotic candidate bacteria obtained from this study to fish.

## Authors’ Contributions

MM and AI: Designed the study. MM, AI, and IWTW: Methodology. AI and AML: Data collection. AI and MM: Validation. MM, AI, IWTW, and AML: Formal analysis. AI, IWTW, and AML: Investigation. AI and AML: Data curation. MM, AI, and AML: Writing-original draft preparation. AI, AML, and IWTW: Review and editing. All authors have read and approved the final manuscript.
